# Everybody's Page

**Published:** 1888-07-14

**Authors:** 


### Everybody's Page.

A 2 per cent. solution of carbolic acid, sprayed lightly about the bed-covering, is said to be a certain preventive of trouble from gnats and mosquitos.

The success of the anti-vaccinators is aptly shown by the results in Zurich, where for a number of years, till 1883, a compulsory vaccination law was in force, and small-pox was wholly prevented, not a single case having occurred in 1882. This result was seized upon in the following year by the anti-vaccinators, and used against the necessity for any such law; and it seems they had sufficient influence to procure its repeal. The death returns for that year (1883) showed that in every 1,000 deaths 2 were caused by small-pox; in 1884, there were 3 in 1,000; in 1885, 17; and in the first quarter of 1886, 85.

There is a very general dislike to the word nervous, but there is no reason for this antipathy, as nervous people are, in many instances, the very salt of the earth. Used in a medical sense, the term simply means that the individual to whom it is applied is delicately adjusted in every way. Frequently endowed with brilliant imagination, subtle penetration, and quick sympathy, they are the master spirits of art and literature. They are capable of self-denial, and, willing to spend and be spent in the performance of their duties, they have endurance enough to struggle on until a task is done, when complete prostration follows.

Very frequently a cough produces headache. The cough, which is simply an exaggerated and modified form of breathing, increases the pressure upon the great veins which bring the blood back to the heart; this increase of pressure prevents the return of the impure blood from the head, while the heart continues to pump blood thereto, and the result is a rise of pressure. With each fit of coughing there is a fulness of the blood-vessels of the head and neck, with flushing of the face, lividity of the lips, and suffusion of the eyes as symptoms of what is passing inside. With these appearances there is a sharp pain through the head accompanying each attack of the cough.

The *Lady's Pictorial* says that a celebrated London medical authority writes: "I think very few of the English public, nor indeed of the cooks who cater for the public at restaurants, etc., know how excellent a result is attainable in the form of 'aspic' by the proper use of 'Liebig's Extract.' The summer season is essentially the time for consuming it. Nothing is more tempting in appearance, in flavour, and in coolness, than aspic jelly as the medium in which little cold fillets of all kinds—fish, fowl, game, lamb, lobster, etc.—may be served, as well as salads. 'Nelson's Gelatine' as the basis, 'Liebig' to colour and give the meat quality and body a little Tarragon vinegar and Worcester sauce to flavour, and a little lemon juice, give at a ridiculously small cost a perfect aspic jelly."

Through the conduct of the Provisional Government in France at the time of the Revolution in 1789, when the introduction into France of British or Spanish soda was prohibited, a process had to be invented for the purpose of making common washing soda or natron from something else than the ashes of plants, and it was then discovered by a French chemist, Le Blanc, that if you start with common salt you can convert the salt into a body which is halfway between salt and washing soda, and then you can afterwards take that intermediate substance and by a second stage convert the material into washing soda. That process, invented by Le Blanc in France, spread itself to England in 1814, and became, and has continued to be, one of our most important technical industries.

The customary dose of castor oil to the new-born infant is not only unnecessary, but mischievous, if not cruel. It is a piece of unwarrantable cruelty to compel a defenceless infant to commence life with that most nauseous of all drugs.

Children, weak patients, and epileptics should not be left alone in a bath, even a shallow bath, during the fraction of a second. Should a fit or fainting come on, the patient may be ost before any assistance can reach him.

We have not yet heard the last of the miraculous ointment brought before our notice by a Derbyshire correspondent. We published a week or two ago his protest against our incredulity regarding its merits, and the prescription he sent us. He also gave us to understand that it was being used in the Derbyshire General Infirmary, though the general complaint there was that it was so slow in action as to be unsuitable for complaints from which the patient wanted to recover in this world. It seems, however, that the gallant inventor is somewhat under a delusion as to the use his ointment is put to, as the following letter which we have received will show: "Dear Sir,—From a paragraph in The Hospital of last week, it might appear to some that an ointment, of which the prescription is given, is being used as an application to diseased bone in the Derbyshire General Infirmary. I beg to state that this is not the case. A somewhat similar preparation is at present being used for polishing the floors here.—Yours truly, J. Acton Southern, House Surgeon." To what base uses may our great ideas come! The function of our wonderful ointment seems to be, not to save bones, but to bring the ward floors to that state of slippery polish, dear to the housekeeper, which tends to make those who walk on them fall and break their limbs.

What shall we be taught next? Most of us had the impression that on whatever points we needed instruction we could sleep—especially in the morning about the time we ought to be getting up—without being told how. Quite a mistake! We have slept, it is true, but for countless generations the human race has been sleeping inartistically, unscientifically, wrongly in every way; so, at least, says Dr. Menli Hitly, of Buchs, as reported in the Paris "Journal d'Hygiene." We ought to sleep with our heads low, and use our pillows to prop up our feet. There seems a certain topsy-turveydom in this, to the average mind; but, quoth Dr. Menli Hitly, of Buchs, it has never yet been proved that the ordinary attitude of slumber is good. True, oh sapient doctor, but it's comfortable; and besides, many facts exist before they are proved—nay, they even exist although they can't be proved by any data yet ascertained; a fact which some scientists, rashly prone to think they have reached the summit of knowledge, are apt to forget. Our doctor assures us that by sleeping in the attitude he advises we shall lessen the work of the heart and facilitate the reparation of nervous losses. Both of these objects are very desirable, but, unfortunately for the theory of Dr. Menli Hitly, of Buchs, one of the primary conditions of sleep is the withdrawal of blood from the brain rather than the reverse. Rest is impossible without a certain degree of cerebral anæmia, as is proved by the fact that we cannot sleep when over-work or excitement of any kind has distended the vessels of the brain. We don't want too much blood pumped up to our brains when we are sleeping. Besides, people who suffer from any weakness of the heart—for whom, above all, the new method is supposed to be suitable—are usually troubled with a breathlessness which might reach a fatal climax if they slept with their heads down and their feet up. On the whole, we think it better to keep to the old method, with all grateful respect to Dr. Menli Hitly, of Buchs.

